# Cardiometabolic profile of young women with hypoprolactinemia

**DOI:** 10.1007/s12020-022-03145-1

**Published:** 2022-07-29

**Authors:** Robert Krysiak, Karolina Kowalcze, Bogusław Okopień

**Affiliations:** 1 Department of Internal Medicine and Clinical Pharmacology, Medical University of Silesia, Katowice, Poland; 2 Department of Pediatrics in Bytom, School of Health Sciences in Katowice, Medical University of Silesia, Katowice, Poland

**Keywords:** Cardiovascular risk factors, Dopamine agonists, Insulin sensitivity, Prolactin deficiency

## Abstract

**Purpose:**

Unlike hyperprolactinemia, clinical significance of prolactin deficiency remains poorly understood. The aim of this study was to assess the cardiometabolic profile of patients with low prolactin levels.

**Methods:**

The study population consisted of three groups of young women. Two groups were chronically treated with cabergoline but differed in prolactin levels, which were either abnormally low (group A; *n* = 16) or within the reference range (group B, *n* = 23). Group C, serving as a control group, included 28 drug-naïve women with normal prolactin levels. The dose of cabergoline in group A was then tapered down. Glucose homeostasis markers, plasma lipids and circulating levels of hormones, uric acid, high-sensitivity C-reactive protein (hsCRP), fibrinogen and homocysteine, as well as the carotid intima-media thickness were assessed at baseline and 6 months later.

**Results:**

Compared with subjects with normal prolactin levels, women with hypoprolactinemia had higher levels of 2-h postchallenge glucose, glycated hemoglobin, triglycerides, uric acid, hsCRP and fibrinogen, lower values of HDL-cholesterol, total testosterone and free androgen index, as well as reduced insulin sensitivity. No differences in these variables were observed between groups B and C. Apart from prolactin normalization, cabergoline dose reduction reversed all laboratory disturbances reported in group A.

**Conclusion:**

The obtained results suggest that hypoprolactinemia in women of reproductive age may increase cardiometabolic risk.

## Introduction

Chronic prolactin excess is associated with increased risk of insulin resistance, prediabetes, atherogenic dyslipidemia, obesity, overweight, subclinical atherosclerosis and impaired endothelial function [1–4]. Unfavorable cardiometabolic effects of hyperprolactinemia were reversed by dopaminergic agents, particularly by cabergoline [5, 6]. Moreover, untreated hyperprolactinemia was found to attenuate the impact of hypolipidemic agents on circulating levels of cardiometabolic risk factors: statins in young women with isolated hypercholesterolemia [7] and fibrates in young women with atherogenic dyslipidemia [8].

Much less is known about clinical manifestations of abnormally low levels of this hormone. Prolactin deficiency results in a failure of lactation (puerperal alactogenesis) and at least in rodents impairs physiological ovulation and produces multiple reproductive defects [9]. Recently, Krysiak et al. reported that iatrogenic hypoprolactinemia impairs sexual functioning and reduces wellbeing in both premenopausal women [10] and young and middle-aged men [11]. Finally, in males consulted for sexual dysfunction, hypoprolactinemia, defined as prolactin levels in the lowest quartile, was associated with a higher prevalence of metabolic syndrome [12]. This paucity of data encouraged us to investigate whether the cardiometabolic profile of patients with low prolactin levels differs from that observed in individuals with prolactin levels within the reference range.

## Materials and methods

### Study population

We studied two groups of women (18 to 45 years old) treated for at least 6 months with cabergoline (0.5–2 mg weekly) because of previous hyperprolactinemia. Group A included 16 subjects with hypoprolactinemia, defined as circulating prolactin levels less than 5 ng/mL. In turn, group B included 23 individuals with prolactin levels within the reference range (5–25 ng/mL). Women in whom despite cabergoline treatment, prolactin levels were still above the upper limit of normal (*n* = 8), were not included. Both groups were compared with 28 age-matched drug-naïve women with normal prolactin levels (group C). In order to minimize the impact of seasonal fluctuations in the outcome variables, 35 women (8 in group A, 12 in group B and 15 in group C) were recruited between January and March, while the remaining ones between July and September. Forty-eight patients included in the present study participated also in the previous one [10].

The remaining exclusion criteria were as follows: diabetes, thyroid disorders or other endocrine disorders (except for prolactinoma); impaired renal or hepatic function; cardiovascular disease (except for mild arterial hypertension); any other serious disorders; pregnancy or lactation; and any chronic pharmacological treatment (with the exception of cabergoline).

All procedures were conducted in line with the Declaration of Helsinki. The study protocol was approved by the institutional review board, and written informed consent was provided by all study patients.

### Study design

In order to normalize prolactin levels, the doses of cabergoline in group A were then decreased by 25%. In group B, the drug was administered at the same doses as before the beginning of the study, while group C did not receive any drugs. Drug adherence was measured every 6 weeks by pill count. The participants were also required to comply with the goals of lifestyle modification. Compliance with non-pharmacological recommendations was measured by analysis of individual dietary questionnaires and of diaries in which the participants recorded all their activities.

### Measurements

The body mass index and the fat-free mass index were calculated, while the waist circumference and blood pressure were measured as previously described [13]. The measurement of carotid intima-media thickness (CIMT) was performed on the common carotid arteries approximately 1.5 cm proximal to the flow divider using high resolution ultrasonography (Toshiba Aplio-500).

Venous blood samples were taken between 8.00 and 9.00 a.m. after a 12-h overnight fast in a quiet, temperature-controlled room (24–25 °C), at the beginning of the study and six months later. Glucose levels were additionally measured in samples collected 2 h after consumption of 75 g of glucose dissolved in 250 ml water. All samples were collected between days 2 and 5 of the menstrual cycle after the patient had been resting in the seated position for at least 30 min. All assays were conducted in duplicate to ensure reproducibility. Plasma levels of glucose, creatinine, lipids, uric acid and albumin, and whole blood levels of glycated hemoglobin were measured using the multi-analyzer COBAS Integra 400 Plus (Roche Diagnostics, Basel, Switzerland). Plasma levels of insulin, prolactin, total testosterone, estradiol, homocysteine and sex hormone-binding globulin were assayed by direct chemiluminescence using acridinium ester technology (ADVIA Centaur XP Immunoassay System, Siemens Healthcare Diagnostics, Munich, Germany). Plasma levels of high-sensitivity C-reactive protein (hsCRP) were measured by immunoassay with chemiluminescent detection (Immulite 2000XPi, Siemens Healthcare, Warsaw, Poland). Fibrinogen was assessed by the method of Clauss, using an automated BCS XP analyzer (Siemens Healthcare, Warsaw, Poland). The estimated glomerular filtration rate was calculated from creatinine levels using the abbreviated Modification of Diet in Renal Disease study equation. The homeostasis model assessment 1 of insulin resistance (HOMA1-IR) was calculated as fasting serum insulin (mU/L) × fasting blood glucose (mmol/L)/22.5. The HOMA2-IR index was obtained by the program HOMA Calculator v2.2.2. The quantitative insulin-sensitivity check index (QUICKI) was calculated using the following formula: 1/[log fasting serum insulin (μU/mL) + log fasting plasma glucose (mg/dL)]. The free androgen index (FAI) was calculated according to the following equation: FAI = 100 × total testosterone (nmol/L)/sex hormone‐binding globulin (nmol/L).

### Statistical analysis

Data with skewed distributions were log-transformed to improve normality. Between-group comparisons were analyzed using one-way analysis of covariance followed by Bonferroni post-hoc tests after consideration of age, smoking and blood pressure as potential confounders. Comparisons within the same group of subjects were carried out by Student’s paired *t*-tests Qualitative data were compared using the *χ*^2^ test. Correlations were calculated using Pearson’s correlation coefficient. The result was considered statistically significant if the probability value (p) was less than 0.05.

## Results

The average weekly dose of cabergoline did not differ statistically between groups A and B at entry [1.06 (0.46) vs. 0.95 (0.41) mg (*p* = 0.43)] or during the follow-up 0.80 (0.35) vs.0.95 (0.41) mg (*p* = 0.24) weekly, respectively].

With the exception of fat content, there were no differences between the study groups in terms of age, smoking, body mass index, fat-free mass index, waist circumference and blood pressure (Table 1). Expectedly, at study entry, group A differed from the remaining two groups in plasma prolactin concentrations. Compared with groups B and C, hypoprolactinemic women had higher levels of 2-h postchallenge glucose, glycated hemoglobin, triglycerides, uric acid, hsCRP and fibrinogen, and lower values of total testosterone, FAI and HDL-cholesterol. There were also differences between group A and groups B and C in values of HOMA1-IR, HOMA2-IR and QUICKI. CIMT tended to be higher in group A than in the remaining groups (Table 2).

**Table 1:** Baseline characteristics of the study population

	Group A^1^	Group B^2^	Group C^3^
Number of patients	16	23	28
Age [years; mean (SD)]	31 (8)	31 (7)	32 (7)
Smokers [%]/Number of cigarettes a day [n; mean (SD)]/Duration of smoking [months, mean (SD)]	25/8 (5)/89 (38)	30/9 (6)/86 (32)	29/8 (5)/84 (30)
Body mass index [kg/m^2^; mean (SD)]	24.9 (4.1)	24.1 (3.7)	23.6 (3.2)
Fat free mass index [kg/m^2^; mean (SD)]	17.9 (3.1)	18.2 (2.8)	17.8 (2.6)
Fat [%]	28.0 (5.0)*^#^	24.4 (4.6)	24.6 (4.4)
Waist circumference [cm; mean (SD)]	80 (8)	77 (7)	76 (7)
Systolic blood pressure [mm Hg; mean (SD)]	128 (17)	126 (15)	124 (15)
Diastolic blood pressure [mm Hg; mean (SD)]	72 (7)	73 (7)	72 (6)

*SD* standard deviation

^1^Women with cabergoline-induced hypoprolactinemia

^2^Cabergoline-treated women with prolactin levels within the reference range

^3^Cabergoline-naïve women with prolactin levels within the reference range

*statistically significant (*p* < 0.05) vs. group B

^#^statistically significant (*p* < 0.05) vs. group C

**Table 2:** Biochemical variables and intima-media complex thickness in the study population

Variable	Group A^1^	Group B^2^	Group C^3^
Prolactin [ng/mL; mean (SD)]			
At baseline	3.2 (1.4)*^#^	15.3 (5.5)	14.2 (5.9)
After 6 months	14.5 (5.3)^&^	15.0 (5.4)	13.2 (5.8)
Total testosterone [nmol/L; mean (SD)]			
At baseline	1.18 (0.44)*^#^	1.61 (0.37)	1.58 (0.32)
After 6 months	1.64 (0.50)^&^	1.67 (0.43)	1.62 (0.39)
FAI [%; mean (SD)]			
At baseline	1.82 (0.44)*^#^	2.82 (0.39)	2.87 (0.34)
After 6 months	2.72 (0.62)^&^	2.83 (0.44)	2.79 (0.35)
Estradiol [pmol/L; mean (SD)]			
At baseline	130 (62)	142 (42)	139 (48)
After 6 months	139 (64)	147 (45)	150 (44)
Fasting glucose [mmol/L, mean (SD)]			
At baseline	5.11 (0.40)	4.92 (0.35)	4.96 (0.31)
After 6 months	4.96 (0.38)	4.86 (0.23)	4.84 (0.28)
2-h post-load glucose [mmol/L, mean (SD)]			
At baseline	7.22 (0.68)*^#^	6.78 (0.64)	6.69 (0.58)
After 6 months	6.55 (0.65)^&^	6.68 (0.70)	6.61 (0.62)
Glycated hemoglobin [%, mean (SD)]			
At baseline	5.2 (0.3)*^#^	5.0 (0.2)	4.9 (0.2)
After 6 months	4.9 (0.3)^&^	5.0 (0.2)	4.9 (0.3)
HOMA1-IR [mean (SD)]			
At baseline	2.0 (0.6)*^#^	1.4 (0.4)	1.3 (0.4)
After 6 months	1.5 (0.6)^&^	1.3 (0.4)	1.4 (0.3)
HOMA2-IR [mean (SD)]			
At baseline	1.15 (0.20)*^#^	0.83 (0.15)	0.77 (0.14)
After 6 months	0.88 (0.19)^&^	0.78 (0.18)	0.79 (0.17)
QUICKI [mean (SD)]			
At baseline	0.344 (0.021)*^#^	0.364 (0.023)	0.368 (0.025)
After 6 months	0.359 (0.020)^&^	0.368 (0.026)	0.367 (0.022)
Total cholesterol [mmol/L; mean (SD)]			
At baseline	4.32 (0.85)	4.39 (0.75)	4.42 (0.80)
After 6 months	4.46 (0.78)	4.51 (0.71)	4.43 (0.65)
HDL-cholesterol [mmol/L; mean (SD)]			
At baseline	1.22 (0.17)*^#^	1.44 (0.20)	1.50 (0.18)
After 6 months	1.47 (0.23)^&^	1.48 (0.24)	1.52 (0.24)
LDL-cholesterol [mmol/L; mean (SD)]			
At baseline	3.02 (0.45)	2.90 (0.39)	2.87 (0.40)
After 6 months	2.95 (0.42)	2.97 (0.44)	2.88 (0.41)
Triglycerides [mmol/L; mean (SD)]			
At baseline	2.08 (0.53)*^#^	1.57 (0.39)	1.60 (0.40)
After 6 months	1.70 (0.50)^&^	1.62 (0.48)	1.55 (0.43)
Uric acid [mg/dL; mean (SD)]			
At baseline	5.2 (1.5)*^#^	4.2 (1.1)	4.0 (1.2)
After 6 months	4.2 (1.2)^&^	4.1 (1.3)	4.0 (1.4)
hsCRP [mg/L; mean (SD)]			
At baseline	3.2 (0.8)*^#^	2.4 (0.6)	2.5 (0.7)
After 6 months	2.4 (0.7)^&^	2.2 (0.7)	2.3 (0.8)
Fibrinogen [mg/dL; mean (SD)]			
At baseline	385 (87)*^#^	328 (74)	315 (69)
After 6 months	320 (80)^&^	340 (78)	325 (62)
Homocysteine [μmol/L; mean (SD)]			
At baseline	19 (8)	16 (6)	16 (5)
After 6 months	16 (5)	17 (5)	15 (4)
Estimated glomerular filtration rate [ml/min/1.73m^2^; mean (SD)]			
At baseline	94 (16)	92 (15)	98 (16)
After 6 months	96 (18)	97 (19)	95 (18)
CIMT [mm; mean (SD)]			
At baseline	0.64 (0.07)^$^^	0.60 (0.06)	0.60 (0.07)
After 6 months	0.61 (0.06)	0.61 (0.05)	0.59 (0.06)

*CIMT* carotid intima-media thickness, *FAI* free androgen index, *HDL* high-density lipoprotein, *HOMA* homeostasis model assessment of insulin resistance, *IU* international unit, *LDL* low-density lipoprotein, *QUICKI* quantitative insulin-sensitivity check index, *SD* standard deviation

^1^Women with cabergoline-induced hypoprolactinemia

^2^Cabergoline-treated women with prolactin levels within the reference range

^3^cabergoline-naïve women with prolactin levels within the reference range

*Statistically significant (*p* < 0.05) vs. group B

^#^Statistically significant (*p* < 0.05) vs. group C

^&^Statistically significant difference (*p* < 0.05) between post-treatment and baseline values within the same group

^$^*p* = 0.0636 vs. group B

^^^*p* = 0.0754 vs. group C

There were no differences between baseline and follow-up body mass index, fat-free mass index, fat content, waist circumference and blood pressure (data not shown). Apart from prolactin normalization, cabergoline dose reduction decreased postchallenge glucose levels, glycated hemoglobin, triglycerides, uric acid, hsCRP and fibrinogen, increased total testosterone, FAI and HDL-cholesterol, well as normalized all markers of insulin sensitivity. In the remaining groups, there were no differences between baseline and follow-up values of the assessed variables. At the end of the study, the study groups did not differ in prolactin, total testosterone, FAI, estradiol, fasting and post-challenge glucose, markers of insulin sensitivity, plasma lipids, uric acid, hsCRP, fibrinogen and homocysteine, as well as in CIMT (Table 2).

In group A, prolactin levels correlated with 2-h postchallenge glucose (*r* = −0.352; *p* = 0.026), HOMA1-IR (*r* = −0.412; *p* = 0.004), HOMA2-IR (*r* = −0.482; *p* = 0.001), QUICKI (*r* = 0.392; *p* = 0.007), glycated hemoglobin (*r* = −0.341; *p* = 0.029), HDL-cholesterol (*r* = 0.406; *p* = 0.005), triglycerides (*r* = −0.385; *p* = 0.011), uric acid (*r* = −0.425; *p* = 0.002), hsCRP (*r* = −0.467; *p* = 0.001), fibrinogen (*r* = −0.371; *p* = 0.008), total testosterone (*r* = 0.408; *p* = 0.008), FAI (*r* = 0.420; *p* = 0.004) and CIMT (*r* = −0.314; *p* = 0.041). Moreover, there were correlations between changes in prolactin levels during the follow-up in group A and the changes in 2-h postchallenge glucose (*r* = 0.322; *p* = 0.038), HOMA1-IR (*r* = 0.384; *p* = 0.010), HOMA2-IR (*r* = 0.404; *p* = 0.008), QUICKI (*r* = 0.292; *p* = 0.046), glycated hemoglobin (*r* = 0.374; *p* = 0.017), HDL-cholesterol (*r* = 0.305; *p* = 0.040), uric acid (*r* = 0.411; *p* = 0.004), hsCRP (*r* = 0.408; *p* = 0.006), total testosterone (*r* = 0.367; *p* = 0.018) and FAI (*r* = 0.388; *p* = 0.011).

## Discussion

The present study shows for the first time that prolactin deficiency is associated with increased levels of cardiometabolic risk factors. Although hypoprolactinemia resulted from cabergoline treatment, the abnormal cardiometabolic profile of women with prolactin failure seems to be a consequence of acquired lactotroph hypofunction and cannot be attributed to specific properties of this drug. Firstly, the weekly dose of cabergoline did not differ between both groups of cabergoline-treated patients. Secondly, there were no correlations between concentrations of cardiometabolic risk factors and cabergoline dose. Thirdly, there were no differences in glucose homeostasis markers, plasma lipids, uric acid, hsCRP, fibrinogen and homocysteine between patients treated and untreated with cabergoline if baseline circulating levels of this hormone were within the reference range. Fourthly, after normalization of the hormone levels, the metabolic profile did not differ from that observed in drug-naïve apparently healthy young women. Finally, the degree of increase in prolactin levels correlated with the improvement in circulating levels of all assessed variables.

In the current study, hypoprolactinemia was also accompanied by discrete changes in CIMT, which is a significant marker of early atherosclerosis, directly related to cardiovascular morbidity and mortality, independently of conventional risk factors [14, 15]. The increase, which did not reach the level of clinical significance, was observed only at baseline but not at follow-up. Small differences between women with low and normal prolactin levels may be explained by the fact that the study population included subjects who did not reach the age at which atherosclerosis becomes symptomatic in the general female population. However, our findings may suggest that atherosclerotic lesions develop earlier in subjects with subnormal prolactin levels than in their peers, as well as that atherogenesis in subjects with prolactin deficiency may be slowed down if circulating levels of this hormone return back to the reference range.

The obtained results are a bit surprising in light of increased cardiometabolic risk in subjects with elevated prolactin levels [1–4] and beneficial cardiometabolic effects of prolactin-lowering agents [5, 6]. They are, however, in line with observations by Corona et al. [12], and seem to indicate that the relationship between prolactin concentration and cardiometabolic risk at least in women of reproductive age is inversely U-shaped. This means that young females with both abnormally low and abnormally high prolactin production may be more prone to cardiovascular and metabolic complications later in life and overtreatment of hyperprolactinemia does not seem to reduce the risk of cardiometabolic complications associated with prolactin excess. The obtained results allow us to draw also other conclusions. The study by Corona et al. [12] included only men (52.0 ± 12.9 years old), and therefore it is probable that unfavorable cardiometabolic effects of hypoprolactinemia are not limited exclusively to young women but may be observed in different patient populations. Thus, prolactin deficiency should be taken into consideration in various groups of patients with pituitary failure and apparently idiopathic cardiometabolic disturbances. Moreover, because cardiometabolic abnormalities in hypoprolactinemic women reflect lactotroph hypofunction and depend on its degree, similar relationships may be observed also in individuals with hypoprolactinemia inherited genetically (abnormal lactotroph cell development) or in subjects with prolactin deficiency secondary to a destruction of pituitary tissue (autoimmune damage of lactotrophs or the whole anterior lobe, tumors, Sheehan syndrome, surgery or radiotherapy). Finally, prolactin deficiency may contribute to excess mortality observed in women with panhypopituitarism [16], and therefore at least some patients with this condition may require treatment. As our findings indicate, drug dose reduction may result in cardiometabolic benefits in subjects with drug-induced hypoprolactinemia. However, individuals with organic prolactin deficiency are not treated with dopaminergic agents, as well as fail to respond to dopamine antagonists (metoclopramide or domperidone) [9, 16]. Although these patients may benefit from prolactin replacement therapy, effectiveness and safety of recombinant human prolactin has been investigated only in a few preliminary studies [17, 18]. Therefore, at least presently, high-risk individuals with organic hypoprolactinemia seem to be candidates for treatment with drugs improving insulin sensitivity (metformin) and exerting cardiovascular pleiotropic effects (statins).

Based on our findings we can only hypothesize about the mechanistic link between low prolactin levels and cardiometabolic disturbances. The association between between-group differences and similar differences in total testosterone and FAI suggests that the impact of prolactin deficiency may be, at least in part, mediated by abnormally low testosterone levels. In line with explanation, middle-aged women with cardiovascular disease were characterized by lower serum androgen levels than matched controls [19]. Moreover, in postmenopausal women, Debing et al. [20] observed a significant inverse relationship between free testosterone and the presence of severe atherosclerosis. However, in men treated for hyperprolactinemia, testosterone increased in response to cabergoline treatment, even if they developed hypoprolactinemia [21, 22]. This observation suggests that the relationship between prolactin production and testosterone concentration is determined by sex. At least three mechanisms may contribute to low values of testosterone and FAI in the participants of our study. Firstly, because prolactin was found to increase androgen synthesis in cultured porcine theca cells [23], low testosterone levels and FAI values may be secondary to a direct inhibitory effect of prolactin deficiency on testosterone production. Secondly, they may result from reduced LH production because hypoprolactinemic female rats were characterized by significantly decreased LH response to gonadotropin-releasing hormone [24]. Finally, low testosterone and FAI may be a consequence of enhanced testosterone metabolism. Prolactin was found to inhibit in vivo ovarian aromatase activity [25], and its abnormally low production may increase conversion of androgens to estrogens, with a subsequent decrease of circulating testosterone levels. There are also alternative explanations for increased cardiometabolic risk in hypoprolactinemic women. Undesirable cardiometabolic effects of hypoprolactinemia may be associated with inadequate stimulation of the prolactin receptor *via* which this hormone exerts its impact on pancreatic β-cell mass, glucose and insulin production, lipid metabolism and energy homeostasis [26]. These effects may be also mediated centrally by the impact of lactotroph hypofunction on monoaminergic pathways. These pathways, implicated in the regulation of insulin sensitivity, lipid and glucose production and systemic inflammation, are modulated by prolactin [27] and this regulation may be disturbed in individuals with inadequate prolactin production [28].

Some study shortcomings bear mentioning. Because of a small number of participants, the obtained results should be interpreted with caution. The study measured only surrogates, and therefore our findings cannot be easily translated to clinical outcomes. It cannot be fully excluded that there exist some differences in the cardiometabolic profile between iatrogenic and non-iatrogenic prolactin deficiency. Finally, it is not sure whether the impact of hypoprolactinemia is similar in individuals with cardiovascular disease and/or diabetes, not participating in the study.

To sum up, iatrogenic hypoprolactinemia in women of reproductive age was associated with unfavorable cardiometabolic effects. These undesirable effects were, however, transient and were not observed if prolactin levels during treatment were kept within the reference range. The obtained results suggest that hypoprolactinemia should not be regarded as an unimportant laboratory finding but as a condition that may predispose to cardiometabolic disorders. Larger-scale studies with hard endpoints are, however, required to verify this hypothesis.

## Data Availability

The datasets generated and analyzed during the current study are available from the corresponding author on reasonable request.

## Abbreviations

CIMT: carotid intima-media thickness
FAI: free androgen index
HDL: high-density lipoprotein
HOMA: homeostasis model assessment of insulin resistance
IU: international unit
LDL: low-density lipoprotein
QUICKI: quantitative insulin-sensitivity check index
SD: standard deviation
